# Case Report: Primary aortoduodenal fistula caused by a 30-mm inflammatory abdominal aortic aneurysm

**DOI:** 10.3389/fsurg.2025.1582823

**Published:** 2025-06-27

**Authors:** Kentaro Akabane, Yuta Tajima, Shuji Toyama, Yoshihisa Tamate, Tetsuo Watanabe, Tetsuro Uchida

**Affiliations:** ^1^ Division of Cardiovascular Surgery, Sendai City Hospital, Miyagi, Japan; ^2^ Second Department of Surgery, Yamagata University Faculty of Medicine, Yamagata, Japan

**Keywords:** aortoenteric fistula, primary aortoduodenal fistula, inflammatory abdominal aortic aneurysms, open surgery, endovascular repair

## Abstract

Primary aortoduodenal fistula (PADF) is a rare but fatal condition with a high mortality rate. Among these, an even smaller subset is caused by an inflammatory abdominal aortic aneurysm (IAAA). Controlling hemorrhage and infection is the primary concern for lifesaving treatments. The standard treatment involves radical open surgery, although endovascular surgery is considered depending on the patient's condition and emergency. Currently, the optimal surgical strategy remains controversial. This study describes the surgical management of a rare case with PADF caused by an IAAA, highlighting challenges in treatment. A 71-year-old man was referred to our hospital following a sudden massive melena. Computed tomography revealed PADF caused by a suspected IAAA. Emergency anatomical reconstruction, fistula closure, and omental coverage via laparotomy were subsequently conducted. After the primary surgery, the patient experienced two episodes of hemorrhagic shock due to infection-induced rupture at proximal and right leg anastomosis sites, which were treated with endovascular repair. The patient was discharged 3 months after the initial surgery. However, 1 month after discharge, a pseudoaneurysm was discovered at the proximal anastomosis site caused by re-infection-induced rupture, and extra-anatomical reconstruction was performed. Excessive surgical invasion caused disseminated intravascular coagulation, and the patient died 1 week postoperatively. The prognosis for PADF management remains poor. Endovascular repair for emergent hemostasis is effective; however, the appropriate timing of radical surgery for prosthetic infection risk is unknown. Therefore, accumulating cases to establish the optimal treatment strategy and surgical timing is essential for improving survival rates.

## 1 Introduction

Primary aortoduodenal fistula (PADF) is a rare but fatal condition with a high mortality rate. Most cases are caused by atherosclerotic abdominal aortic aneurysms (AAAs), while fewer result from inflammatory AAAs (IAAA), which are considered less prone to rupture. Controlling hemorrhage and infection is the primary concern for lifesaving treatments, which typically involve radical open surgery, with endovascular surgery considered based on the patient's condition and emergency. Nonetheless, no consensus exists on the optimal surgical strategy. This report presents a rare case of PADF caused by a small IAAA (diameter, 30 mm), which was treated through a multidisciplinary approach, and describes the treatment strategy.

## 2 Case description

**Figure 1:**
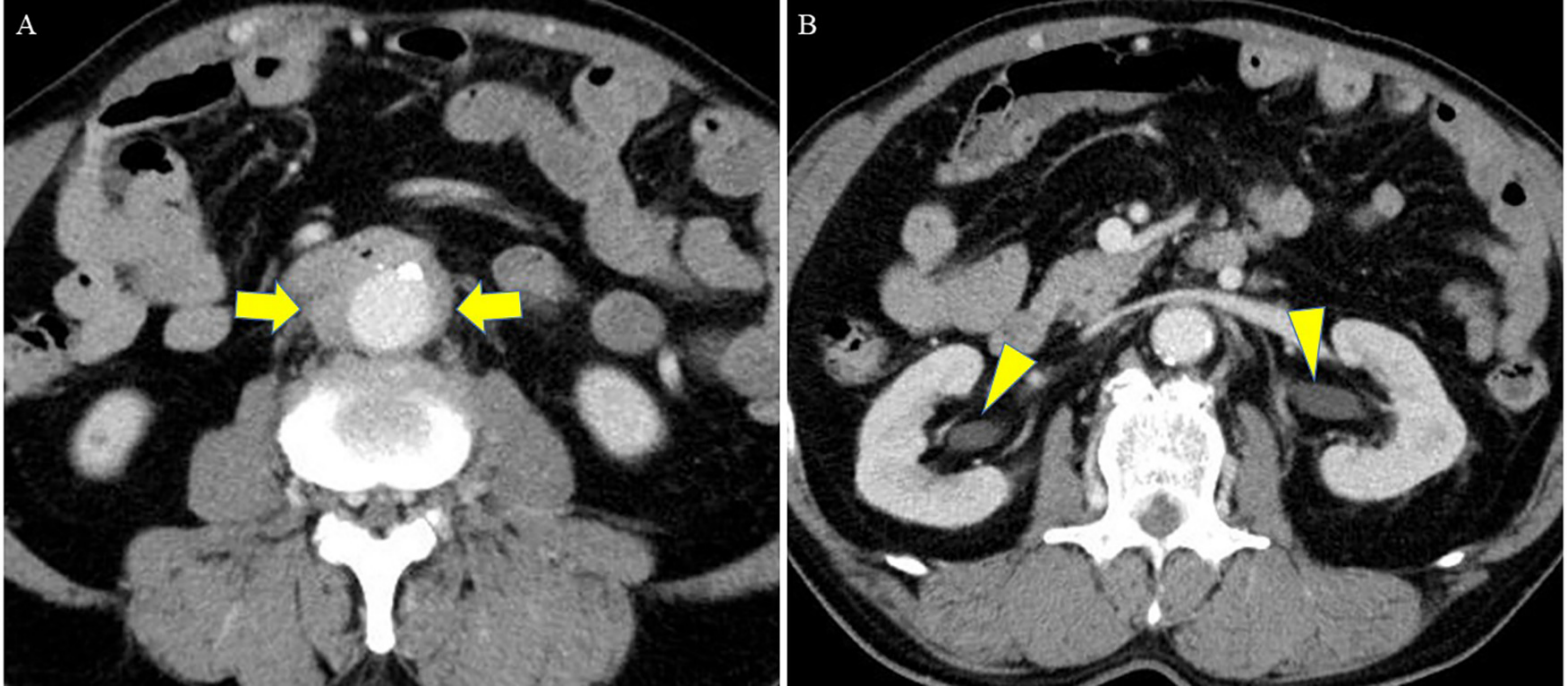
Preoperative CT, **(A)** at the level of the duodenum and **(B)** at the level of the kidney. **(A)** CT revealed an infrarenal fusiform AAA (maximum diameter: 30 mm) with thickening of the surrounding fat tissue. Although no contrast was seen within the intestinal tract, air was detected within the wall of the horizontal segment of the duodenum bordering the anterior surface of the AAA (arrow). **(B)** Bilateral hydronephrosis (arrowhead) and stenosis of the inferior vena cava were detected. CT, computed tomography; AAA, abdominal aortic aneurysm.

A 71-year-old man with no history of cardiovascular or gastrointestinal disorders, no prior surgeries, and no history of smoking was referred to our hospital 1 h after the onset of a sudden massive melena. Upon arrival, the patient's general physical condition was unstable, with a blood pressure of 90/40 mmHg, a heart rate of 120 beats per minute in sinus rhythm, and a Glasgow coma scale score of 12 (E3V4M5). Blood tests showed severe anemia, with a hemoglobin level of 6.7 g/dl. Computed tomography (CT) revealed an infrarenal fusiform AAA, with a maximum diameter of 30 mm and thickening of the surrounding fat tissue. Although no contrast was observed within the intestinal tract, air was detected within the wall of the horizontal segment of the duodenum bordering the anterior surface of the AAA (Figure 1A). Additionally, bilateral hydronephrosis and stenosis of the inferior vena cava were detected (Figure 1B). A diagnosis of PADF, likely caused by an IAAA, was made. As the patient's hemodynamics stabilized with blood transfusions, emergency radical open surgery was planned. Although endovascular aortic repair (EVAR) was considered, open surgery was chosen as a one-stage treatment, considering the infection control, need for duodenum repair, and the uncertainty of EVAR for the unique condition of PADF caused by IAAA.

**Figure 2:**
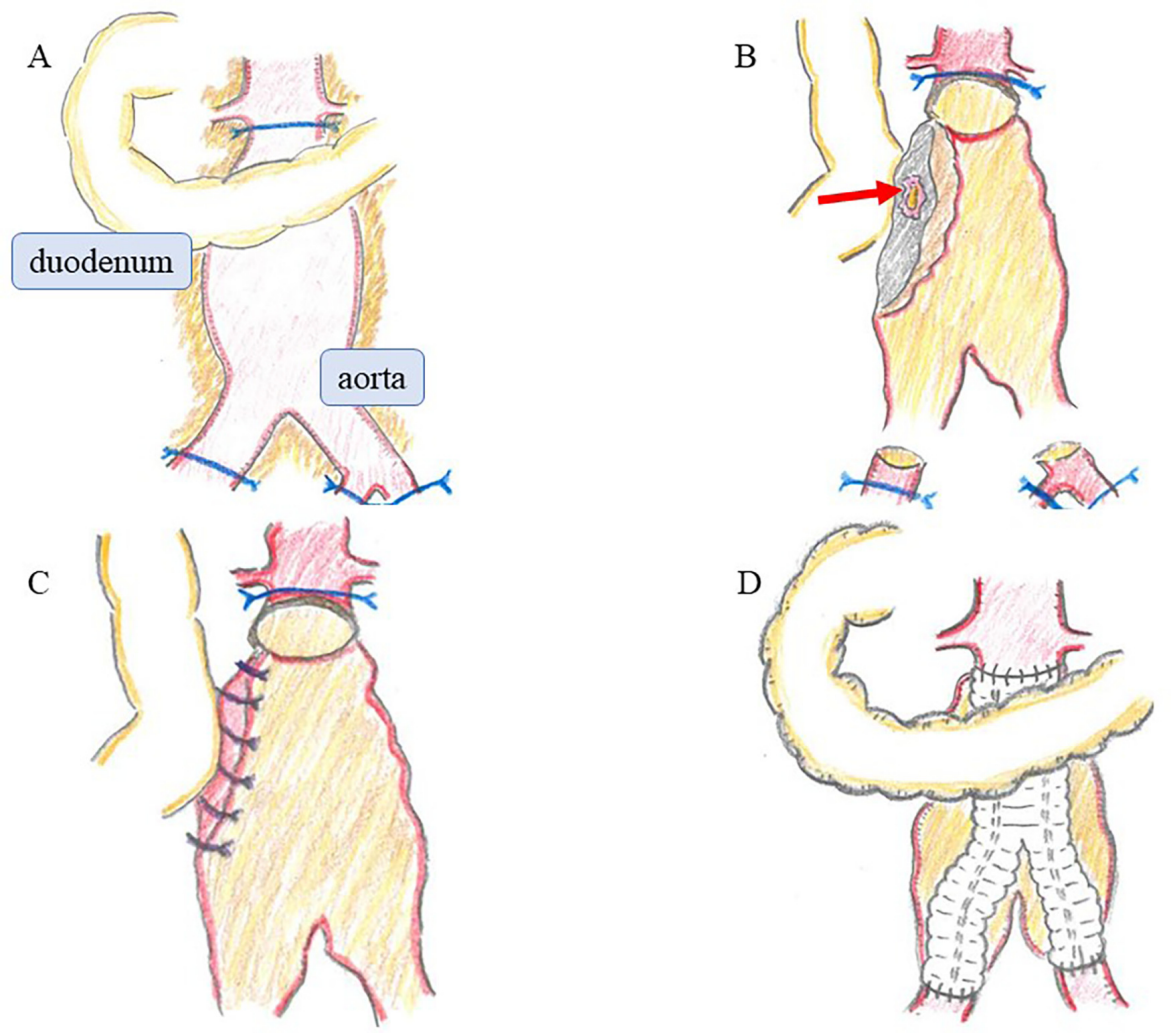
Operative findings. **(A)** The aorta was hard and had a porcelain-like sheen, with dense adhesions to surrounding tissues, consistent with an IAAA. **(B)** Upon opening the aneurysm, a small 1-cm pore leaking intestinal fluid was detected between the intima and adventitia at the area in contact with the third part of the duodenum (arrow). **(C)** The aortic and intestinal walls were sutured together, closing the intestinal fistula and stopping leakage of the intestinal fluid. **(D)** *In situ* aortic reconstruction with prosthetic graft and omental coverage were performed. IAAA, inflammatory abdominal aortic aneurysm.

Under general anesthesia, an emergency laparotomy revealed a significant accumulation of blood within the intestinal tract. The aorta exhibited a hard, porcelain-like sheen with dense adhesions to the surrounding tissues, consistent with the findings of an IAAA (Figure 2A). After the infrarenal aorta, right common iliac artery, and left internal and external iliac arteries were clamped, the aneurysm was opened and inspected from its internal lumen. A small 1-cm pore leaking intestinal fluid was detected between the intima and adventitia at the area in contact with the third part of the duodenum (Figure 2B). Owing to the dense adhesions, separating the duodenum from the surrounding tissue and resecting the fistula site with anastomosis to healthy intestinal tracts was infeasible. Instead, the aortic and intestinal walls were sutured together, closing the intestinal fistula and stopping leakage of the intestinal fluid (Figure 2C). Because of the mild contamination, *in situ* aortic reconstruction with prosthetic graft (Hemashield® GETINGE, Tokyo) and omental coverage were performed (Figure 2D).

**Figure 3:**
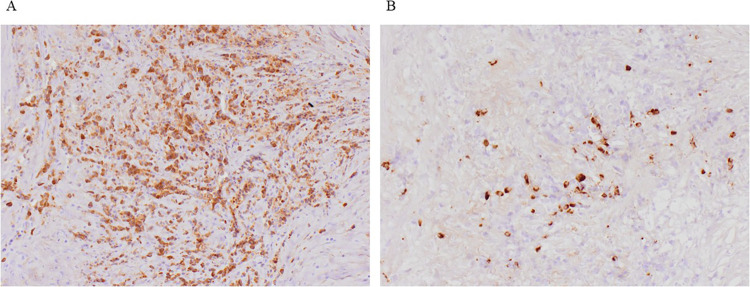
Histopathological findings. **(A)** Histopathological examination showed atherosclerotic changes in the intima, with plasma cell and lymphocyte infiltration in the tunica media and adventitia. Mild perineuritis was also observed, consistent with IAAA. **(B)** Additional immunostaining was performed but the IgG4/IgG positive cell ratio is about 10%, and did not support a diagnosis of immunoglobulin G4-related disease. IAAA, inflammatory abdominal aortic aneurysms.

On postoperative day 8, the patient presented with a high fever exceeding 38°C, and laboratory data also showed a prolonged hyperinflammatory state. CT revealed panperitonitis caused by minor residual intestinal fluid leakage. The patient underwent irrigation and drainage via laparotomy. To prevent exposure of the prosthetic graft to the intestinal fluid, omental coverage was re-applied to separate the graft from the fistula. However, on day 21, the proximal anastomosis site ruptured due to infection, causing hemorrhagic shock. Emergent EVAR was performed (GORE® EXCLUDER® W. L. Gore & Associates, Inc., Newark) to control the bleeding. On day 42, hemorrhagic shock re-occurred due to an infection-induced rupture at the right leg anastomosis site, which was treated with endovascular repair (GORE® VIABAHN®). To prevent a similar complication at the left leg anastomosis site, a stent graft (GORE® VIABAHN® VBX) was prophylactically placed on day 57 to reinforce the anastomosis. The patient resumed oral intake on day 64. Although no organisms were isolated from the fistula area or blood cultures at the time of the initial surgery, *methicillin-resistant Staphylococci* (MRS), *Pseudomonas aeruginosa*, and *Candida species* were subsequently detected in the drained ascitic fluid during the clinical course. Therefore, broad-spectrum antibiotics such as meropenem and tazobactam/piperacillin, anti-MRS agents, and antifungal agents were administered over 2 months. On day 88, the regimen was de-escalated to oral minocycline, and the patient was discharged home on day 94. One month after discharge, CT detected a pseudoaneurysm at the proximal anastomosis site caused by re-infection-induced rupture. Considering massive contamination, extra-anatomical reconstruction was performed, including an aorto-right common iliac artery bypass with the superficial femoral vein, bilateral superficial femoral artery bypass with prosthetic vascular graft, and debridement. However, the excessive surgical invasion led to disseminated intravascular coagulation, and the patient died 1 week postoperatively, 3 months after the initial surgery. Histopathological examination of the aortic tissue obtained during the initial operation showed atherosclerotic changes in the intima, with plasma cell and lymphocyte infiltration in the tunica media and adventitia. Mild perineuritis was also observed, consistent with IAAA (Figure 3A). Additional immunostaining was performed but did not support a diagnosis of immunoglobulin G4-related disease (Figure 3B). At the final surgery, *Candida species* were isolated from the resected perivascular tissue, suggesting that fungal infection may have contributed to the pathogenesis.

## 3 Discussion

Aortoenteric fistulas (AEFs) are rare. Compared to secondary AEFs, which develop after prior prosthetic graft replacement or EVAR, primary AEFs are even rarer, with an incidence rate of 0.04%–0.07% (1). Aorto-duodenal fistulas account for over 80% of AEFs, predominantly affecting the third part of the duodenum (2). The most common cause of PADF is AAA. Given that the duodenum is closely adherent and anterior to the aorta, dilation of the aorta can cause irritation and inflammation, increasing the risk of fistulization (3). Among PADFs, 73% are associated with atherosclerotic aneurysms, while 26% arise from traumatic or mycotic aneurysms (4). Other possible causes include infection, foreign bodies, radiation therapy, and tumors (5). To date, only three cases of PADF associated with IAAA have been reported. Ikeda et al. (6) and Honjo et al. (7) presented cases managed with open surgery, and Suezawa et al. (8) described a case treated with EVAR.

Diagnosing PADFs is often challenging due to their rarity and the non-specificity of their symptoms. The classic triad includes gastrointestinal bleeding (64%–94%), abdominal pain (32%–48%), and abdominal pulsating aneurysm (17%–25%). However, all three symptoms are present in only 11% of the cases (9). Herald bleeding, the first episode of gastrointestinal bleeding, is transient bleeding from a small fistula stopped by vasoconstriction and blood clot formation. In such cases, 30% of the patients experience secondary fatal hemorrhage within the next 6 h (10). In our case, gastrointestinal bleeding was the only symptom of the classic triad. The patient had no history of gastrointestinal bleeding, and his hemodynamics stabilized with blood transfusions, suggesting that this episode could represent herald bleeding. Considering the possibility of subsequent fatal bleeding, emergency surgery was an appropriate decision.

The most useful diagnostic modality is contrast-enhanced CT, which is less invasive than endoscopy or angiography and carries a low risk of thrombus dislodgement. Key CT findings suggest that PADF includes the disappearance of the fat plane between the aneurysm and duodenum, ectopic air in the retroperitoneum or within the aortic wall, and contrast enhancement within the duodenum (11).

Given that the mortality rate of untreated PADF approaches 100%, early diagnosis and prompt surgical treatment are crucial. However, the diagnosis of PADFs is often challenging, and the optimal treatment strategy is not defined, resulting in poor outcomes. Despite surgical intervention, survival rates remain variable (18%–93%), with postoperative complications occurring in up to 40% of patients and an overall postoperative mortality rate exceeding 30% (12). The two primary concerns for lifesaving treatments are controlling hemorrhage at the time of presentation and reducing the risk of late complications associated with bleeding or infection. The important factors in determining the treatment strategy depend on the patient's hemodynamics and comorbidities. For hemodynamically stable patients, the typical surgical procedures include aortic reconstruction and duodenal repair via laparotomy. When contamination is minimal, *in situ* aortic reconstruction using a prosthetic graft with omental coverage is preferred (9). The omental coverage of a prosthetic graft, an independent predictor of survival, is critical for preventing infection (13). In contrast, for cases of PADF with massive contamination, extra-anatomical bypass grafting and extensive debridement are feasible. However, these procedures are associated with high rates of postoperative aortic stump re-infection or disruption, lower limb amputation, and death (14). For patients who are hemodynamically unstable due to gastrointestinal bleeding, EVAR offers a minimally invasive option for closing the fistula and stopping bleeding (15). While EVAR is effective for immediate hemorrhage control, placing a foreign body in the aorta connected to the duodenum poses a high risk of infection. Therefore, EVAR should be considered as a temporary measure to stabilize the patient's condition and serve as a bridge therapy until radical surgery can be performed. However, the optimal timing for radical surgery remains controversial, and no definitive guidelines exist for intervention in PADF (16).

In this case, CT and surgical findings were suspicious of IAAA, and pathological findings confirmed the diagnosis. IAAAs account for 5%–10% of all AAAs (17, 18) and are characterized by a thickened aneurysm wall, significant perianeurysmal and retroperitoneal fibrosis, and dense adhesions to surrounding structures (19). They are more common in men, tend to occur 5–10 years earlier than atherosclerotic AAAs, and have a lower rupture rate (20). The incidence of rupture is 3%–4% in IAAA compared with 17%–20% in non-IAAAs (21, 22). As for treatment strategy, in addition to preventing rupture, the management of impaired organ passage due to fibrous thickening around the aneurysm must be considered. Open surgery can be significantly challenging due to severe periaortic fibrotic adhesions to surrounding tissues, which increase the risk of iatrogenic injuries during adhesiolysis. The 1-year all-cause mortality rate is 14%, compared to 2% for EVAR (23). Thus, in anatomically suitable patients, EVAR should be considered as a first-line treatment option. However, long-term outcomes following EVAR remain to be elucidated, and some studies suggest that stent graft legs may bend and become narrow in cases with significant inflammation and adhesions (24). Additionally, symptoms caused by impaired organ passage, such as hydronephrosis, may improve more effectively with open surgery. Therefore, for patients with longer life expectancy and impaired organ passage, such as hydronephrosis, open surgery may be a preferable option compared with EVAR (25).

We encountered a rare case of PADF due to IAAA, with the smallest aneurysm reported to date, measuring 30 mm in diameter. As the patient's hemodynamics were stabilized by blood transfusion before the first surgery, open surgery was chosen as a one-stage treatment, considering the curative potential of infection control, treatment of the duodenum, and the uncertainty of EVAR for the unique condition of PADF caused by IAAA. During hemorrhagic shock due to anastomotic site rupture, EVAR was performed emergently for hemorrhage control. The patient was discharged, and the endovascular repair was deemed effective for hemostasis. However, shortly after discharge, the patient experienced a re-infection-induced rupture at the anastomosis site and eventually died.

The prognosis for PADF management remains poor, highlighting the need for accumulating cases to determine the optimal treatment strategy and surgical timing to improve survival rates. This case illustrates the critical challenge of achieving durable infection control in PADF associated with IAAA and adds to the limited literature on this rare but life-threatening condition.

## Data Availability

The original contributions presented in the study are included in the article/Supplementary Material, further inquiries can be directed to the corresponding author.
